# Genetic and biological properties of H7N9 avian influenza viruses detected after application of the H7N9 poultry vaccine in China

**DOI:** 10.1371/journal.ppat.1009561

**Published:** 2021-04-27

**Authors:** Xin Yin, Guohua Deng, Xianying Zeng, Pengfei Cui, Yujie Hou, Yanjing Liu, Jingzhen Fang, Shuxin Pan, Dongxue Wang, Xiaohan Chen, Yaping Zhang, Xiurong Wang, Guobin Tian, Yanbing Li, Yan Chen, Liling Liu, Yasuo Suzuki, Yuntao Guan, Chengjun Li, Jianzhong Shi, Hualan Chen

**Affiliations:** 1 State Key Laboratory of Veterinary Biotechnology, Harbin Veterinary Research Institute, CAAS, Harbin, People’s Republic of China; 2 College of Life and Health Sciences, Chubu University, Aichi, Japan; University of Texas Medical Branch / Galveston National Laboratory, UNITED STATES

## Abstract

The H7N9 avian influenza virus (AIV) that emerged in China have caused five waves of human infection. Further human cases have been successfully prevented since September 2017 through the use of an H7N9 vaccine in poultry. However, the H7N9 AIV has not been eradicated from poultry in China, and its evolution remains largely unexplored. In this study, we isolated 19 H7N9 AIVs during surveillance and diagnosis from February 2018 to December 2019, and genetic analysis showed that these viruses have formed two different genotypes. Animal studies indicated that the H7N9 viruses are highly lethal to chicken, cause mild infection in ducks, but have distinct pathotypes in mice. The viruses bound to avian-type receptors with high affinity, but gradually lost their ability to bind to human-type receptors. Importantly, we found that H7N9 AIVs isolated in 2019 were antigenically different from the H7N9 vaccine strain that was used for H7N9 influenza control in poultry, and that replication of these viruses cannot, therefore, be completely prevented in vaccinated chickens. We further revealed that two amino acid mutations at positions 135 and 160 in the HA protein added two glycosylation sites and facilitated the escape of the H7N9 viruses from the vaccine-induced immunity. Our study provides important insights into H7N9 virus evolution and control.

## Author summary

Human infection with H7N9 virus has been successfully prevented since the application of an H7N9 vaccine in poultry in September 2017 in China; however, the H7N9 virus has not been eradicated from poultry. In this study, we evaluated the genetic and biologic properties of H7N9 viruses detected in poultry in China from February 2018 to December 2019. We found that the H7N9 viruses gradually lost their binding to human-type receptors and were antigenically different from the H7N9 vaccine strain that was used in China for H7N9 influenza control in poultry. We further uncovered the genetic changes that facilitate the escape of the H7N9 viruses from vaccine-induced immunity. Our study provides important insights into H7N9 virus evolution and control.

## Introduction

H7N9 influenza viruses emerged in China in early 2013 and caused five waves of human infections from 2013–2017, with a total of 1568 cases, of which 615 were fatal [1]. The early H7N9 viruses were low pathogenic and did not cause disease in avian species [2–4]. After four years of circulation in nature, however, some low pathogenic H7N9 strains acquired an insertion of four amino acids in their hemagglutinin (HA) cleavage site in early 2017 and became highly pathogenic in chickens, causing severe influenza outbreaks in poultry in China [5–7]. These highly pathogenic H7N9 viruses posed an increased threat to human health given their pandemic potential and virulence to humans [7–9].

To control H7N9 influenza virus, an H5/H7 bivalent inactivated vaccine was initiated in China in September 2017. The H5 seed virus (Re-8) contains the HA and NA genes from the clade 2.3.4.4 virus A/chicken/Guizhou/4/2013 (H5N1) and its six internal genes from the high-growth A/Puerto Rico/8/1934 (H1N1) (PR8) virus and has been used in China since 2015 [7, 10], and the H7 seed virus (H7-Re1) is a reassortant bearing the HA and NA genes of the H7N9 low pathogenic virus A/pigeon/Shanghai/1069/2013 and the six internal genes of PR8 [11]. The vaccine was updated and an H5/H7 trivalent inactivated vaccine had been used since December 2018 [12]. The vaccine was developed by using the Re-11, Re-12, and H7-Re2 vaccine seed viruses, which were generated by reverse genetics and derived their HA genes from A/duck/Guizhou/S4184/2017 (H5N6) (a clade 2.3.4.4d virus) (Re-11), A/chicken/Liaoning/SD007/2017 (H5N1) (a clade 2.3.2.1d virus) (Re-12), and A/chicken/Guangxi/SD098/2017 (H7N9), respectively [12]. Large scale surveillance performed before and after the H5/H7 inactivated vaccine application indicated that the vaccination program dramatically reduced the prevalence of H7N9 virus in poultry [7]. More importantly, the vaccination of chickens successfully prevented human infections with H7N9 virus as indicated by the fact that there were 766 human cases reported between October 1, 2016, and September 30, 2017, but only three and one human case over the same time period of the following two years [13, 14], and no human case has been detected since March 2019 [1, 15] (S1 Fig).

Although the prevalence of H7N9 virus in poultry has declined considerably and human infection has been essentially eliminated since the application of the H7N9 poultry vaccine, the H7N9 influenza virus has not been eradicated from poultry; it has caused a few disease outbreaks in chickens and zoo birds in northern China in 2018 and 2019 [16]. When vaccines are used to control influenza virus in the field, the potential emergence of new strains that can evade vaccine-induced immunity is always a concern. To monitor for such strains, we carried out active surveillance in poultry in China from February 2018 to December 2019. We isolated 19 H7N9 viruses and analyzed their genetics, virulence in different animals, receptor-binding properties, and antigenicity. Our findings provide important insights into the evolution of H7N9 influenza viruses since the application of the H7N9 vaccine in poultry in China.

## Results

### Isolation and genetic analysis of H7N9 viruses

In total, 19 H7N9 AIVs were isolated: 13 H7N9 AIVs were isolated from 71,018 samples that were collected in China from February 2018 to December 2019 during our routine surveillance, and six viruses were isolated from the samples that were submitted to our laboratory for disease diagnosis (Table 1). The virus-positive samples were from two provinces in southern China (Anhui and Fujian) and six provinces in northern China (Shaanxi, Shanxi, Inner Mongolia, Ningxia, Hebei, and Liaoning). Twelve viruses were isolated from layer chickens, five viruses were isolated from broilers, one virus was isolated from a duck, and one virus was isolated from a peacock in a zoo. The diagnosis samples were from unvaccinated chickens, ducks, and zoo birds; however, we do not know the vaccination background of the birds we sampled during surveillance.

**Table 1:** H7N9 viruses isolated between February 2018 and December 2019.

Virus		Sample information				
Full name	Abbreviation	Date	Province	Location	Source	Avian species
A/chicken/Anhui/SE0105/2018	CK/AH/SE0105/18	February 2018	Anhui	Poultry market	Surveillance	Broiler
A/chicken/Anhui/SE0296/2018	CK/AH/SE0296/18	February 2018	Anhui	Poultry market	Surveillance	Broiler
A/duck/Fujian/SE0377/2018	DK/FJ/SE0377/18	February 2018	Fujian	Poultry market	Surveillance	Duck
A/chicken/Liaoning/SD003/2018	CK/LN/SD003/18	February 2018	Liaoning	Farm	Surveillance	Broiler
A/chicken/Shaanxi/SD004/2018	CK/SaX/SD004/18	March 2018	Shaanxi	Farm	Diagnosis	Layer
A/chicken/Anhui/S1032/2018	CK/AH/S1032/18	March 2018	Anhui	Poultry market	Surveillance	Broiler
A/chicken/Shanxi/SD006/2018	CK/SX/SD006/18	April 2018	Shanxi	Farm	Diagnosis	Layer
A/chicken/Ningxia/SD007/2018	CK/NX/SD007/18	April 2018	Ningxia	Farm	Diagnosis	Layer
A/chicken/Ningxia/SD008/2018	CK/NX/SD008/18	May 2018	Ningxia	Farm	Diagnosis	Layer
A/chicken/Liaoning/SD009/2018	CK/LN/SD009/18	May 2018	Liaoning	Farm	Diagnosis	Layer
A/chicken/Hebei/SD010/2018	CK/HeB/SD010/18	October 2018	Hebei	Farm	Surveillance	Layer
A/chicken/Liaoning/SD014/2018	CK/LN/SD014/18	December 2018	Liaoning	Farm	Surveillance	Layer
A/peacock/Liaoning/SD004/2019	PCK/LN/SD004/19	March 2019	Liaoning	Zoo	Diagnosis	Peacock
A/chicken/Inner Mongolia/SD010/2019	CK/IM/SD010/19	April 2019	Inner Mongolia	Poultry market	Surveillance	Broiler
A/chicken/Hebei/S1118/2019	CK/HeB/S1118/19	April 2019	Hebei	Slaughterhouse	Surveillance	Layer
A/chicken/Hebei/S1140/2019	CK/HeB/S1140/19	April 2019	Hebei	Slaughterhouse	Surveillance	Layer
A/chicken/Hebei/S1177/2019	CK/HeB/S1177/19	April 2019	Hebei	Slaughterhouse	Surveillance	Layer
A/chicken/Liaoning/SD025/2019	CK/LN/SD025/19	November 2019	Liaoning	Farm	Surveillance	Layer
A/chicken/Liaoning/SD026/2019	CK/LN/SD026/19	December 2019	Liaoning	Farm	Surveillance	Layer

To investigate their genetic relationship, the whole genomes of the 19 H7N9 AIVs were fully sequenced (the sequence data have been deposited in the Global Initiative on Sharing Avian Influenza Data; the accession numbers are EPI1853780~EPI1853931). All 19 AIVs had a similar amino acid motif of -PKRKRTAR/G- at their HA cleavage site, which meets the criterion for highly pathogenic AIVs [17]; the viruses are therefore referred to as highly pathogenic AIVs (HPAIVs) throughout this manuscript. Previous studies have shown that the amino acids 186V and 226L in HA (H3 numbering used throughout) are important for the H7N9 viruses to bind human-type receptors; 94.2% and 97.6% of the H7N9 low pathogenic AIVs isolated between 2013 and 2017 have 186V and 226L, respectively, in their HA gene [6]. Our sequences analysis revealed that all 19 HPAIVs in this study have 186V but 226Q in their HA (S1 Table).

**Fig 1:**
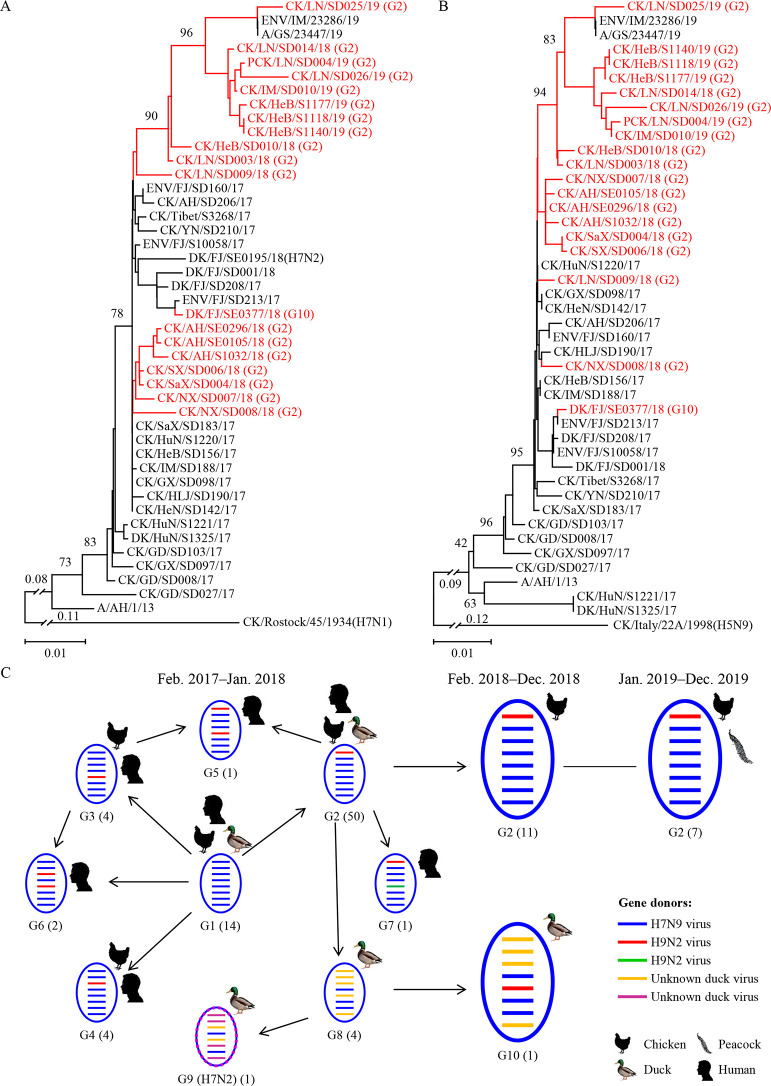
Phylogenetic analyses and genotypes of H7N9 highly pathogenic avian influenza viruses. The phylogenetic trees of the HA **(A)** and NA **(B)** genes were rooted to A/chicken/Rostock/45/1934 (H7N1) and A/chicken/Italy/22A/1998 (H5N9), respectively. The viruses sequenced in this study are shown in red in the phylogenetic trees. **(C)** Genotypes of H7N9 and H7N2 viruses and the hosts in which these genotypes were detected. The genotypes of the viruses isolated between February 2017 and January 2018 were reported previously [7]; the viruses isolated between February 2018 and December 2019 were analyzed in this study. The numbers of strains of each genotype are provided in parentheses.

The HA gene of the 19 H7N9 viruses share 97%–100%; NA gene 97.6%–100%, and matrix (M) gene 97.1%–100% identity at the nucleotide level; they cluster in phylogenetic trees with the H7N9 HPAIVs that were previously detected in 2017 (Figs 1A and 1B and S4A). The basic polymerase 2 (PB2), basic polymerase 1 (PB1), acidic polymerase (PA), nucleoprotein (NP), and nonstructural protein (NS) genes of 18 of the 19 viruses are closely related, sharing 97.6%–100%, 98%–100%, 98%–100%, 98%–100%, and 97.7%–100% identity at the nucleotide level, respectively, but they are quite different from the DK/FJ/SE0377/18 virus, sharing only 85.7%–86.2%, 88.8%–89.4%, 90.5%–91.4%, 93.7%–94.6%, and 69.1%–69.7% identity, respectively. The PB2, PB1, PA, NP, and NS of DK/FJ/SE0377/18 and the other 18 viruses locate in different groups in the phylogenetic trees (S2A, S2B, S3A, S3B and S4B Figs).

We previously reported that the H7N9 HPAIVs isolated in China between February 2017 and January 2018 formed nine genotypes [7]. On the basis of their genomic similarity, the 19 H7N9 HPAIVs in this study were divided into two genotypes: 18 of the 19 viruses belong to the genotype 2 that was reported previously by Shi et al. [7], whereas DK/FJ/SE0377/18 formed a different genotype, likely as a reassortant of the previously reported genotype 8 virus and an H9N2 virus [7]; we therefore assigned this virus to genotype 10 (G10) (Fig 1C).

### Virulence of H7N9 highly pathogenic avian influenza viruses in mice

**Fig 2:**
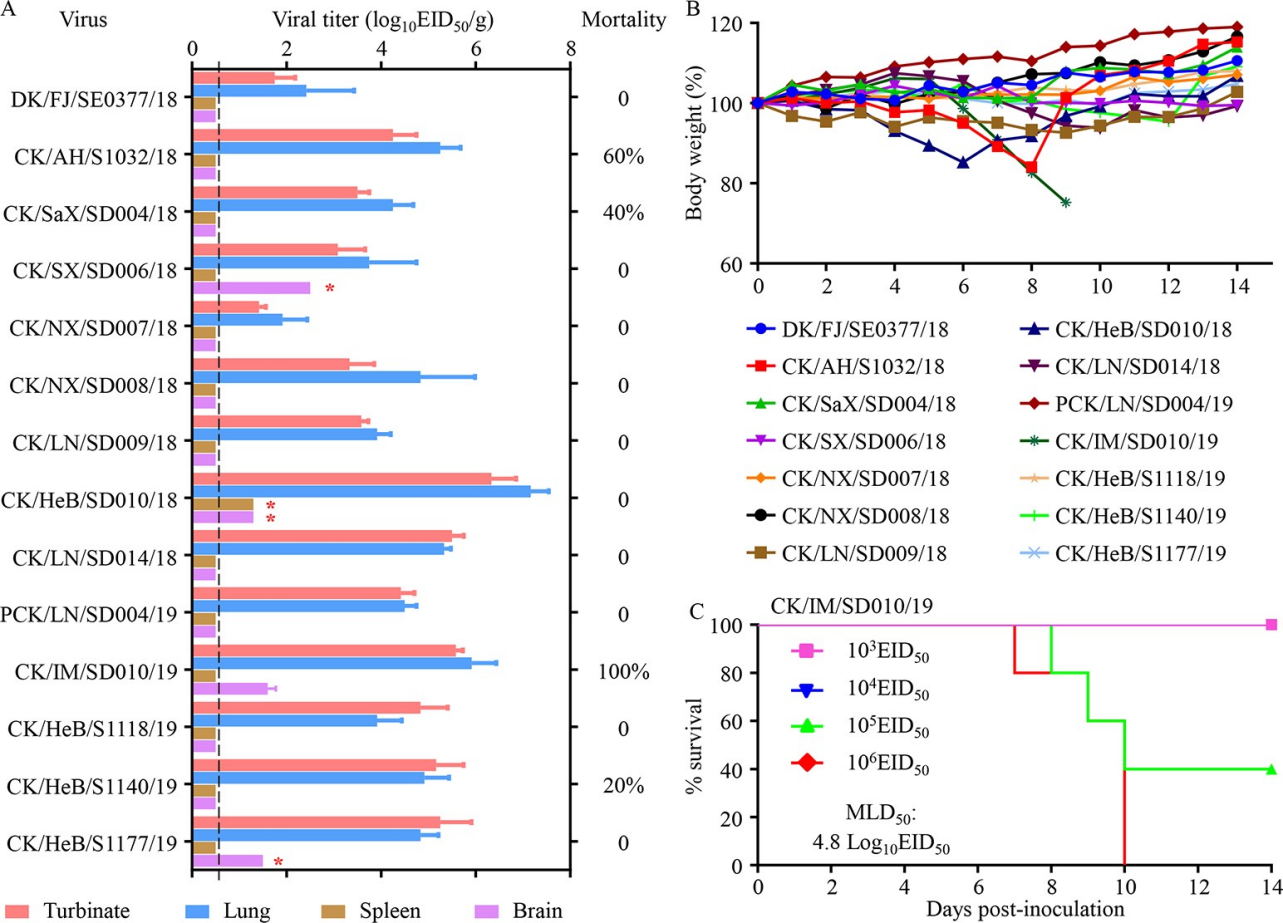
Replication and virulence of H7N9 viruses in mice. **(A)** Viral titers in organs of mice after inoculation with 10^6^ EID_50_ of different viruses. Three mice from each group were killed on Day 3 p.i., and virus titers were determined in eggs. Color bars show the mean, and the error bars represent standard deviations. The values labeled with a red star indicate that the virus was only detected in the organ of one mouse. The dashed lines indicate the lower limit of virus detection. **(B)** Changes in body weight in the groups of five mice after inoculation with 10^6^ EID_50_ of different viruses. **(C)** Mouse-lethal doses of the CK/IM/SD010/19 virus.

Previous studies reported that H7N9 HPAIVs isolated from chickens in 2017 have different virulence in mice [6, 7]. To understand the virulence of the recent H7N9 HPAIVs, we tested 14 H7N9 representative strains that were isolated at different times, from different locations, and from different avian species. Groups of eight mice were intranasally inoculated with 10^6^EID_50_ of the test viruses; three mice in each group were euthanized on Day 3 post-inoculation (p.i.) to assess virus replication in their organs, and the remaining five mice were observed for body weight changes and death for up to two weeks. All 14 viruses replicated in the nasal turbinates and lungs of all three mice tested; four viruses were also detected in the brain of one mouse, and one virus was detected in the spleen of one mouse, but virus was not detected in the kidneys of any mice (Fig 2A). Ten of the viruses were not lethal during the two-week observation period; however, CK/HeB/S1140/19, CK/SaX/SD004/18, and CK/AH/S1032/18 killed one, two, and three of the five mice, respectively, whereas CK/IM/SD010/19 killed all five mice (Fig 2A). The surviving mice lost less than 16% of their body weight (Fig 2B). We then tested the 50% mouse lethal dose (MLD_50_) of CK/IM/SD010/19 by inoculating groups of five mice i.n. with 10^3.0^–10^6.0^ EID_50_ of the virus and found its MLD_50_ to be 4.8 log_10_EID_50_ (Fig 2C). These results indicate that the H7N9 HPAIVs have distinct virulence in mice, although most of them are non-lethal to mice.

### Virulence of the H7N9 viruses in chickens and ducks

The H7N9 viruses detected in this study have an HA cleavage site that meets the criterion for HPAIVs, yet some of them were isolated from apparently normal poultry during routine surveillance. To investigate their replication and virulence in poultry, we tested three representative viruses isolated from different hosts (CK/LN/SD009/18, DK/FJ/SE0377/18, and PCK/LN/SD004/19) in chickens and ducks.

Groups of 11 six-week-old specific pathogen-free (SPF) chickens were inoculated i.n. with 10^6^ EID_50_ of these viruses. Pharyngeal and cloacal swabs were collected from all birds on Day 3 p.i.; three chickens in each group were then killed and their organs (lungs, heart, liver, spleen, kidneys, pancreas, and brain) were collected for virus titration in eggs, and the remaining eight chickens were observed for signs of disease and death. The H7N9 viruses were detected in both the pharyngeal and cloacal swabs, and replicated systemically in the chickens, with high titers detected in all of the tested organs. The remaining eight chickens in each group died within 5 days of inoculation (Table 2).

**Table 2:** Replication and virulence of H7N9 viruses in chickens and ducks^a^.

Avian species	Virus	Virus shedding on Day 3 post inoculation (p.i.): positive/total (mean titer, log_10_ EID_50_/ml)		Virus replication in organs on Day 3 p.i.: positive/total (mean titer, log_10_ EID_50_/g)							Death/ Total	Sero-conversion: positive/total (HI titer range)
		Pharynx	Cloacae	Lung	Heart	Liver	Spleen	Kidney	Pancreas	Brain		
Chicken	CK/LN/SD009/18	11/11 (7.0)	11/11 (4.3)	3/3 (7.8)	3/3 (6.8)	3/3 (6.1)	3/3 (6.3)	3/3 (7.3)	3/3 (6.5)	3/3 (6.1)	8/8	/
	DK/FJ/SE0377/18	11/11 (5.8)	11/11 (4.3)	3/3 (7.0)	3/3 (6.6)	3/3 (5.0)	3/3 (6.0)	3/3 (6.4)	3/3 (5.3)	3/3 (5.9)	8/8	/
	PCK/LN/SD004/19	11/11 (4.9)	11/11 (4.5)	3/3 (6.1)	3/3 (5.7)	3/3 (4.3)	3/3 (5.1)	3/3 (5.5)	3/3 (4.5)	3/3 (5.1)	8/8	/
Duck	CK/LN/SD009/18	1/8 (1.3)	<	<	<	<	<	<	<	<	0/5	0/5
	DK/FJ/SE0377/18	5/8 (2.1)	1/8 (1.3)	3/3 (2.9)	3/3 (2.8)	1/3 (3.5)	3/3 (1.8)	1/3 (3.5)	<	<	0/5	5/5 (8–32)
	PCK/LN/SD004/19	6/8 (1.8)	1/8 (1.8)	2/3 (2.3)	<	<	<	1/3 (1.3)	<	<	0/5	5/5 (32–128)

a Groups of 11 six-week-old specific-pathogen-free chickens and groups of eight three-week-old specific-pathogen-free ducks were inoculated i.n. with 10^6^ EID_50_ of each virus in a 0.1-ml volume. Pharyngeal and cloacal swabs were collected from all birds on Day 3 p.i., and then three birds in each group were euthanized, and their organs were collected for virus titration in eggs. The remaining eight chickens or five ducks in each group were observed for two weeks. <, virus was not detected from the undiluted samples. /, all birds in that group died before the scheduled antiserum collection at two weeks after virus inoculation.

Groups of eight three-week-old SPF ducks were inoculated i.n. with 10^6^ EID_50_ of these viruses. Pharyngeal and cloacal swabs were collected from all birds on Day 3 p.i. Three ducks in each group were then killed and their organs (lungs, heart, liver, spleen, kidneys, pancreas, and brain) were collected for virus titration in eggs; the remaining five ducks were observed for signs of disease and death. The CK/LN/SD009/18 virus was detected in the pharyngeal swab of one of the eight ducks, but not in the cloacal swab or any organs tested; all five ducks survived the infection, and none of them seroconverted (Table 2). The DK/FJ/SE0377/18 virus was detected in the pharyngeal swab and cloacal swab of five ducks and one duck, respectively; it was also detected in the liver and kidneys of one duck and in the lungs, heart, and spleen of all three ducks that were euthanized on Day 3 p.i.; all five ducks survived their infections and seroconverted (Table 2). The PCK/LN/SD004/19 virus was detected in the pharyngeal swab and cloacal swab of six ducks and one duck, respectively; it was also detected in the lungs and kidneys of two ducks and one duck, respectively, that were euthanized on Day 3 p.i.; all five ducks survived their infections and seroconverted (Table 2). These results indicate that the H7N9 viruses are highly lethal in chickens but cause mild infection in ducks.

### Receptor-binding properties of the H7N9 viruses

**Fig 3:**
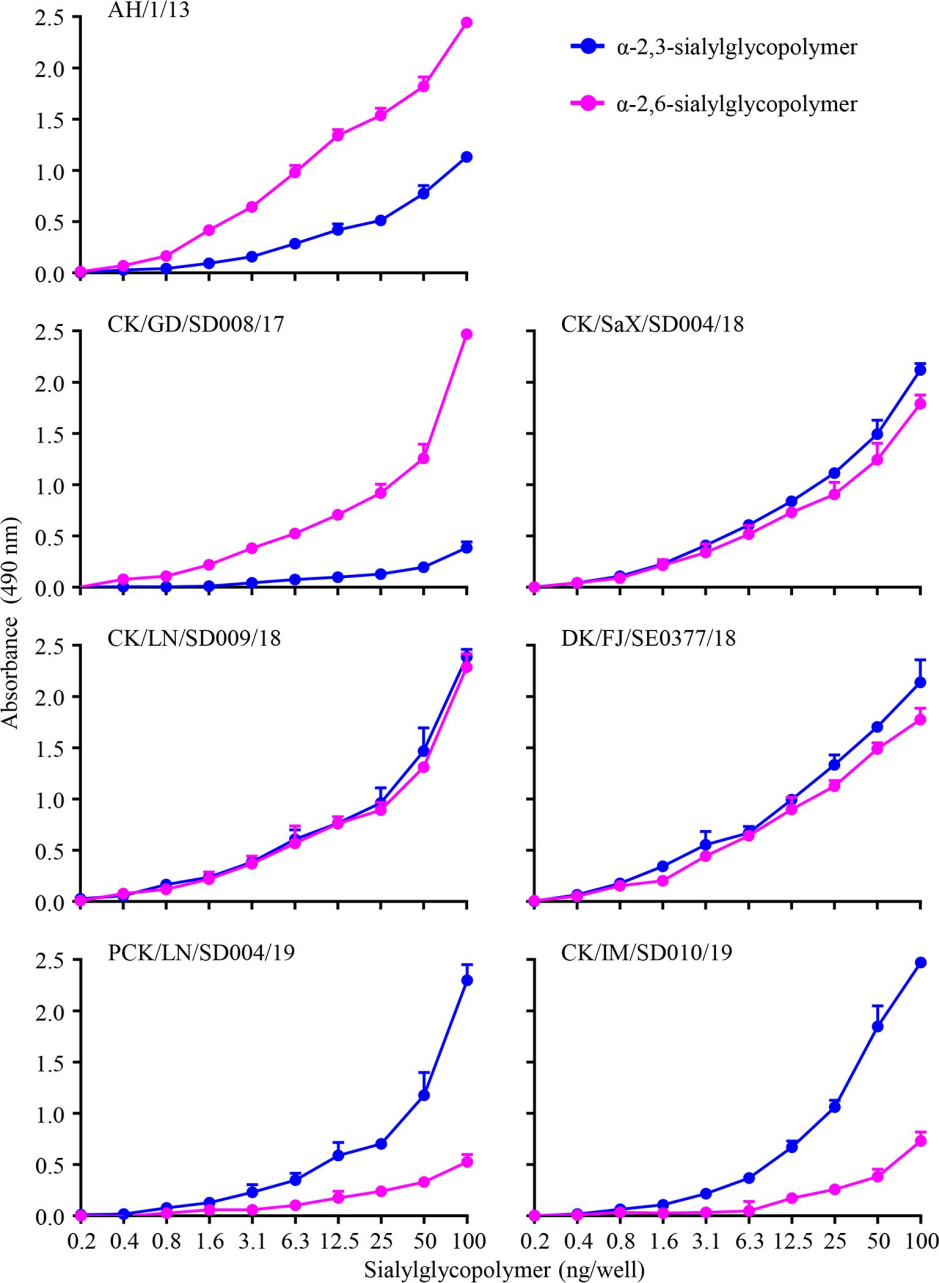
Receptor-binding properties of H7N9 representative viruses isolated between 2013 and 2019. The binding of H7N9 viruses to two different glycans (α-2,3-glycans, blue; α-2,6-glycans, pink) was assessed. The data shown are the means of three repeats, the error bars indicate standard deviations.

Previous studies have shown that H7N9 low pathogenic viruses bind to human-type receptors with high affinity, and two amino acids (186V and 226L) in their HA contribute to this property [6]. The amino acid 186V is conserved in all H7N9 HPAIVs; however, 226L in HA has only been detected in a few early H7N9 HPAIVs [6, 7]. All 19 H7N9 viruses we isolated in this study bear 226Q in their HA (S1 Table). To investigate the receptor-binding properties of the H7N9 HPAIVs, we selected five strains (three from 2018 and two from 2019) and tested their affinity for two different glycopolymers: α-2, 3-sialylglycopolymer [Neu5Acα2-3Galb1-4GlcNAcb1-pAP (para-aminophenyl)-alpha-polyglutamic acid (α-PGA)] (avian-type receptor) and α-2, 6-sialylglycopolymer [Neu5Acα2-6Galb1-4GlcNAcb1-pAP (para-aminophenyl)-alpha-polyglutamic acid (α-PGA)] (human-type receptor). We also included two early viruses––A/Anhui/1/13 (H7N9) (AH/1/13) and the index H7N9 HPAIV A/chicken/Guangdong/SD008/2017 (H7N9) (CK/GD/SD008/17)––in this analysis for comparison. AH/1/13 and CK/SD008/17 bound to the α-2, 6-sialylglycopolymer with high affinity and to the α-2, 3-sialylglycopolymer with low affinity, the three viruses isolated in 2018 bound to the α-2, 3-sialylglycopolymer and α-2, 6-sialylglycopolymer with similar affinities), whereas the two viruses isolated in 2019 bound to the α-2, 3-sialylglycopolymer with high affinity and to the α-2, 6-sialylglycopolymer with low affinity (Fig 3). These results indicate that H7N9 HPAIVs have different receptor-binding properties and that the viruses isolated in 2019 have weakened ability to bind to human-type receptors.

### Antigenic variation of H7N9 HPAIVs

**Fig 4:**
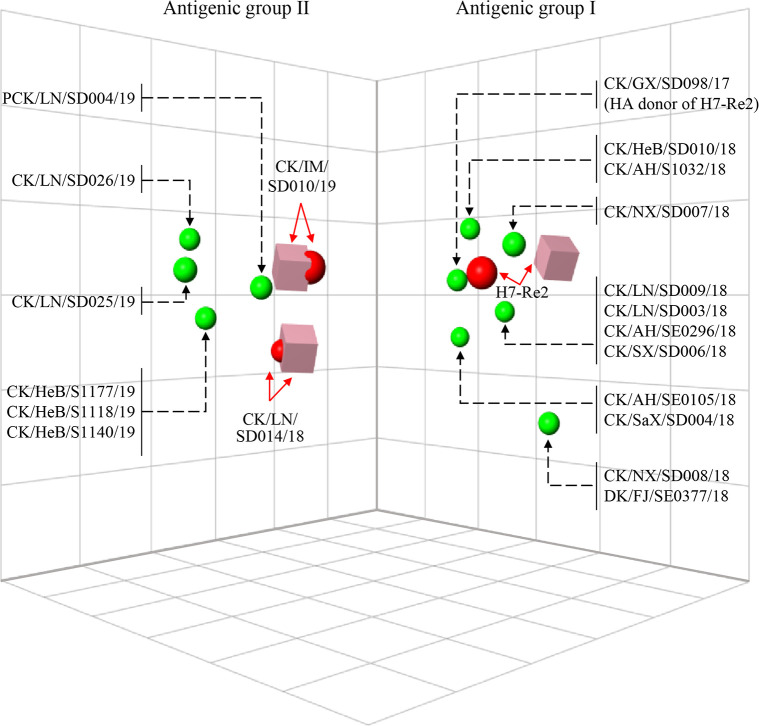
Antigenic cartography of H7N9 viruses. The antigenic map was generated by using the HI assay data shown in S2 Table. Each unit in the coordinate represents a 2-fold difference in HI titer. The pink cubes represent the antisera generated from the indicated viruses. The red balls indicate the viruses used for antisera generation, and the green balls show the test viruses.

Influenza viruses easily undergo antigenic variation when they circulate in nature. Since we began controlling H7N9 influenza in poultry through vaccination, monitoring the antigenic variation of newly detected viruses is a priority for avian influenza surveillance. To investigate the antigenic properties of the H7N9 HPAIVs, chicken antisera were generated against the H7-Re2 vaccine strain and two H7N9 strains (CK/LN/SD014/18 and CK/IM/SD010/19) that were isolated in this study. The cross-reactivity of these three antisera against the vaccine strain H7-Re2, A/chicken/Guangxi/SD098/2017 (H7N9) (CK/GX/SD098/17) (H7-Re2 HA donor virus) [12], and the 19 H7N9 HPAIVs isolated in this study were evaluated by using the hemagglutination inhibition (HI) test. We found that 11 viruses isolated in 2018 cross-reacted well with the H7-Re2 antiserum, and their HI antibody titers were 2–4 fold lower than that to the homologous viruses H7-Re2 and CK/GX/SD098/17; the other eight viruses, including one virus isolated in December 2018 and seven viruses isolated in 2019, reacted poorly with the H7-Re2 antiserum, and their HI antibody titers were 8–32 fold lower than that to the homologous viruses H7-Re2 and CK/GX/SD098/17 (S2 Table). H7-Re2, CK/GX/SD098/17, and all seven viruses that were isolated in 2019 cross-reacted well with the CK/LN/SD014/18 antiserum, and their HI antibody titers were 2–4 fold lower than that to the homologous virus, but the other 11 viruses that were isolated in 2018 cross-reacted poorly with the CK/LN/SD014/2018 antiserum, and their HI antibody titers were 8-fold lower than that to the homologous virus (S2 Table). H7-Re2, CK/GX/SD098/17, and 17 of the 19 viruses analyzed in this study cross-reacted well with the CK/IM/SD010/19 antiserum, but the HI antibody titers of two viruses isolated in 2018 (DK/FJ/SE0377/18 and CK/NX/SD008/18) were 16-fold lower than that to the homologous virus (S2 Table). The HI data were further analyzed quantitatively by means of antigenic cartography [18], and these viruses formed two different antigenic groups: the first group (antigenic group I) contained the vaccine seed virus H7-Re2, CK/GX/SD098/17, and 11 viruses isolated in 2018, and the second group (antigenic group II) contained one virus isolated in 2018 and seven viruses isolated in 2019 (Fig 4). These results indicate that the H7N9 viruses isolated in 2019 are antigenically different from the H7-Re2 vaccine strain.

### Protective efficacy of the H5/H7 trivalent inactivated vaccine in chickens against different H7N9 viruses

An H5/H7 trivalent inactivated vaccine developed by using two H5 seed viruses (Re-11 and Re-12) and one H7N9 seed virus (H7-Re2) has been used in poultry since December 2018 [12]. Does the H5/H7 trivalent inactivated vaccine provide complete protection against the antigenically drifted H7N9 viruses? To answer this question, we selected five viruses, including two viruses from antigenic group I (CK/GX/SD098/17 and CK/SX/SD006/18) and three viruses from antigenic group II (PCK/LN/SD004/19, CK/IM/SD010/19, and CK/LN/SD25/19), and performed challenge studies in chickens.

**Fig 5:**
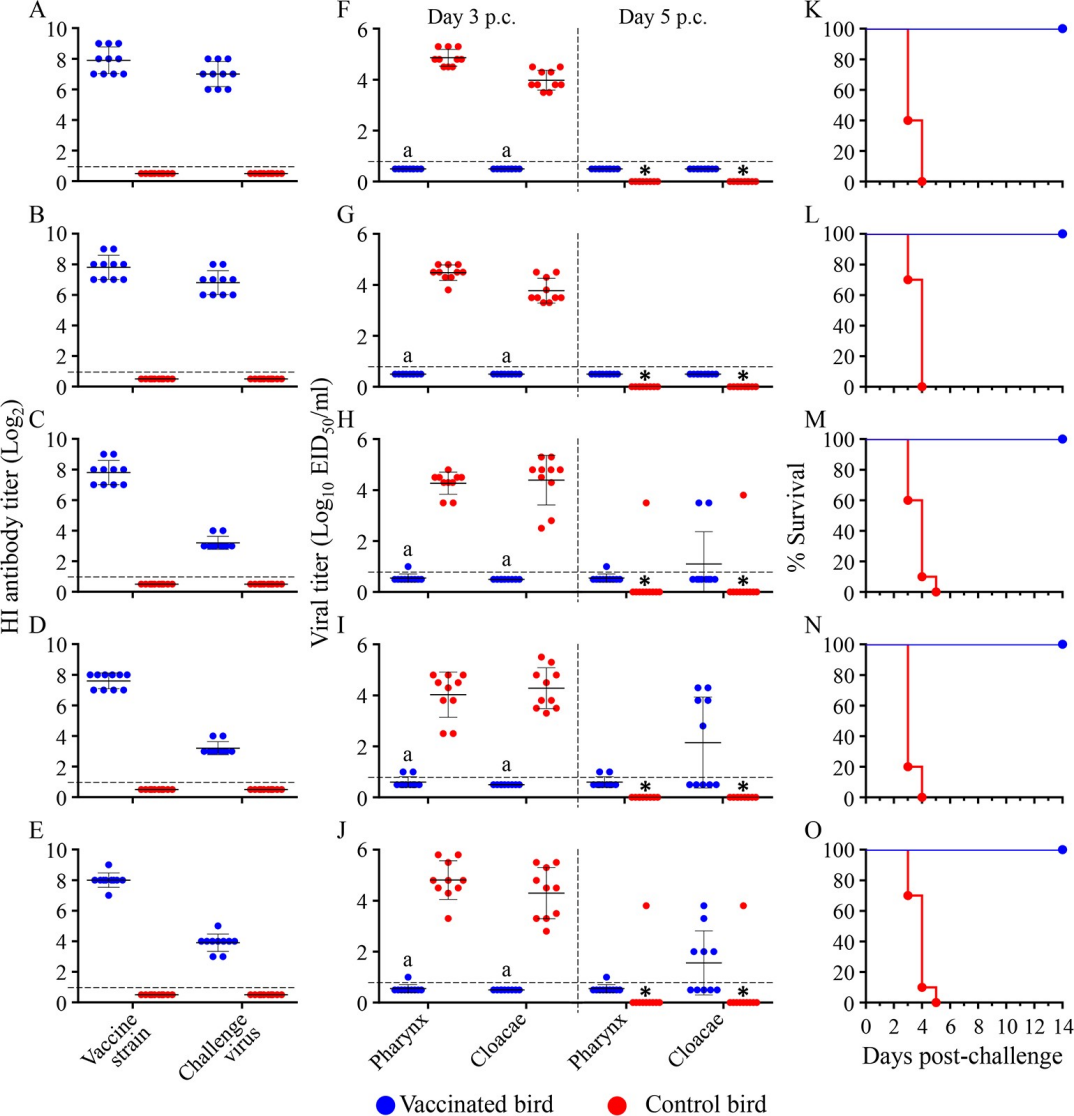
Protective efficacy of H5/H7-Re2 trivalent inactivated vaccine against challenge with different H7N9 viruses in chickens. HI antibody titers (**A-E**), virus shedding titers **(F-J)**, and survival patterns **(K-O)** of chickens challenged with the H7N9 highly pathogenic viruses CK/GX/SD098/17 **(A, F, and K)**, CK/SX/SD006/18 **(B, G, and L)**, PCK/LN/SD004/19 **(C, H, and M)**, CK/IM/SD010/19 **(D, I, and N)**, and CK/LN/SD25/19 **(E, J, and O)**. The dashed lines shown in **A-E** show the cutoff value for seroconversion and those in **F-J** show the lower limit of virus detection. Virus titers shown in **F-J** are the means from the birds that survived. A value of 0.5 was assigned to virus shedding-negative birds for statistical purposes. The asterisks indicate that the bird(s) died before that day, and therefore virus shedding data were not available for statistical analysis. All of the chickens in these control groups died within 5 days of challenge. The letter “a” indicates *p* < 0.001 compared with the corresponding titers of the control birds.

Groups of 10 three-week-old female SPF chickens were inoculated intramuscularly with one dose of 0.3 ml of the vaccine or with an equal volume of PBS as a control. Sera were collected for antibody detection and chickens were challenged with different H7N9 viruses at three weeks post-vaccination. The mean HI antibody titers in the five groups of vaccinated chickens ranged from 7.8 log2 to 8.3 log2 against the H7-Re2 vaccine strain, and were 7.0 log2, 6.8 log2, 3.2 log2, 3.2 log2, and 3.9 log2 against CK/GX/SD098/17, CK/SX/SD006/18, PCK/LN/SD004/19, CK/IM/SD010/19, and CK/LN/SD25/19, respectively (Fig 5A–5E). Chickens in the control groups did not have detectable antibodies against these viruses (Fig 5A–5E).

After challenge with the H7N9 HPAIVs, all of the control chickens shed viruses through both the pharynx and cloacae and died within 5 days of challenge (Fig 5F–5O). Virus shedding was not detected at any timepoints from any vaccinated chickens that were challenged with CK/GX/SD098/17 or CK/SX/SD006/18 (Fig 5F and 5G); however, two of the 10 vaccinated chickens that were challenged with PCK/LN/SD004/19, and five of the 10 vaccinated chickens that were challenged with CK/IM/SD010/19 or CK/LN/SD25/19 shed virus (Fig 5H–5J). All of the vaccinated chickens were apparently healthy and survived for the duration of the two-week observation period post-challenge (Fig 5K–5O). These results indicate that the H5/H7-Re2 trivalent vaccine was immunogenic and provided full protection in chickens against challenges with H7N9 viruses of antigenic group I, but was unable to completely prevent the replication of H7N9 viruses of antigenic group II.

### Molecular basis for the antigenic difference in H7N9 viruses

**Fig 6:**
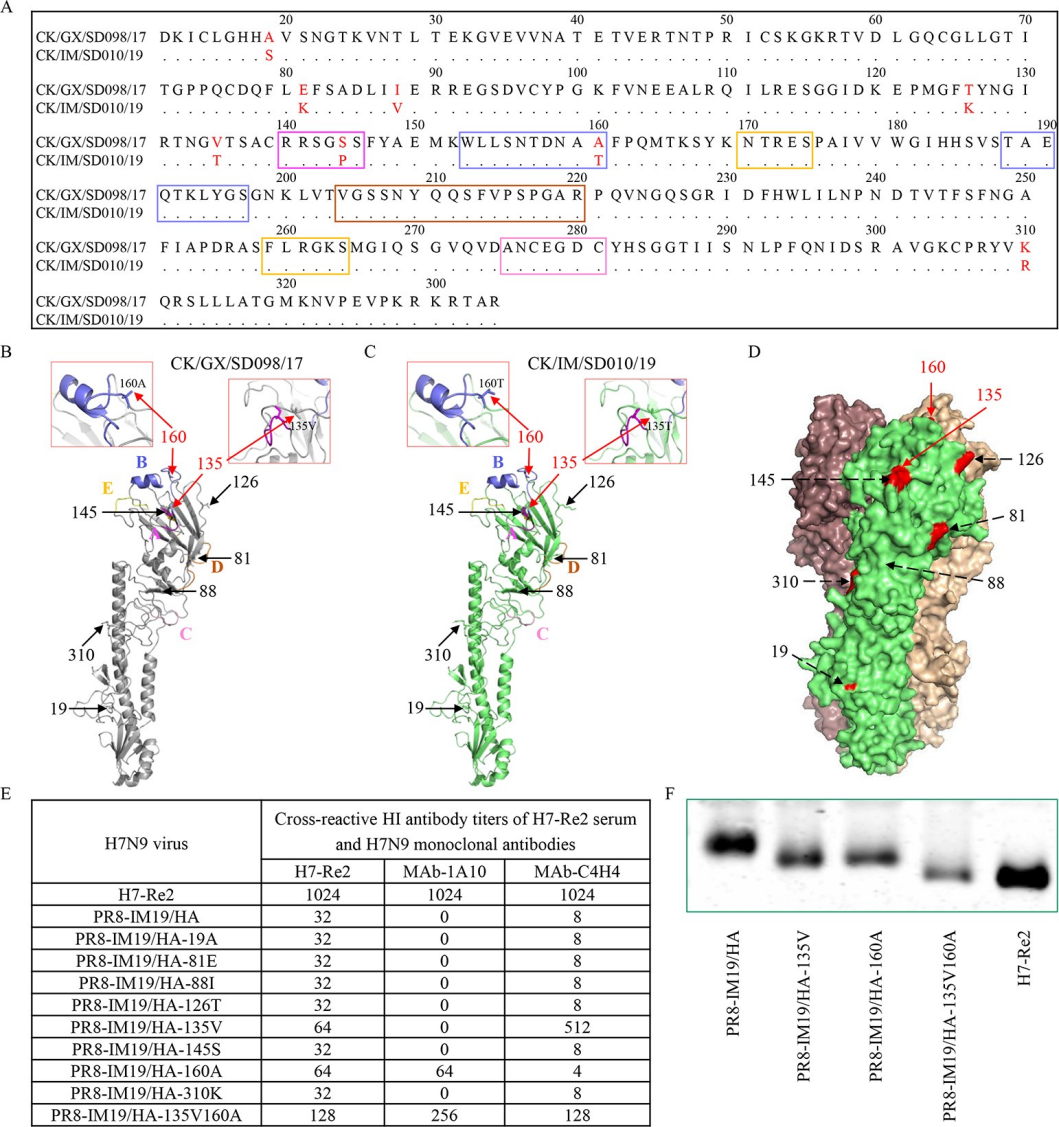
Key mutations in HA that contributed to the antigenic drift of the 2019 H7N9 viruses. **(A)** Amino acid differences in the HA1 protein of the representative H7N9 viruses CK/GX/SD098/17 and CK/IM/SD010/19. The key amino acids in the head of the HA1 trimer that differ between the two viruses are shown in red. The colored boxes show different antigenic regions (site A to site E). The 2D structure of the HA1 protein of CK/GX/SD098/17 **(B)** and CK/IM/SD010/19 **(C),** and the 3D structure of the HA1 protein of CK/GX/SD098/17 **(D)** were obtained by using SWISS-MODEL; images were drawn with Pymol software. The numbers show the positions of the key amino acid in the head of the HA1 trimer that are different in the representative viruses. **(E)** HI titers of different H7N9 mutants against H7-Re2 antiserum and H7N9 monoclonal antibodies. **(F)** Mobility of H7N9 avian influenza HA1 protein analyzed by SDS-PAGE and Western blotting.

To investigate the molecular basis of H7N9 virus antigenic drift, we compared the HA gene and identified eight amino acids in the HA protein that were conserved in most of the viruses in antigenic group I, but mutated in viruses in antigenic group II (S3 Table and Fig 6A). Seven of these eight amino acids, except for the one at position 88, are located on the surface of the globular head of the HA1 protein, as shown in the simulated 2D structure of the HA1 protein of CK/GX/SD098/17 and CK/IM/SD010/19 (Fig 6B and 6C) or the 3D structure of CK/GX/SD098/17 (Fig 6D), which we generated by using SWISS-MODEL (www.swissmodel.expasy.org) [19, 20] and Pymol software. To pinpoint which amino acid residue contributes to the antigenic drift, we generated a reassortant bearing the six internal genes of the PR8 virus and the HA and NA genes of CK/IM/SD010/19; of note, four amino acids in the HA cleavage site were deleted to make the resultant virus low pathogenic. This virus was designated PR8-IM19/HA. PR8-IM19/HA cross-reacted with the antiserum of H7-Re2 with titers 32-fold lower than that to the homologous virus (Fig 6E). We also tested the reactivity of PR8-IM19/HA with two monoclonal antibodies (MAbs), MAb-1A10 and MAb-C4H4, that were generated against chicken/Shanghai/S1053/2013 (H7N9), and found that PR8-IM19/HA did not react with MAb-1A10, but did react with MAb-C4H4 with low titers (Fig 6E).

Next, we generated eight mutants in the PR8-IM19/HA background, each of which contained one amino acid change as shown in Fig 6E. Six mutants responded to H7-Re2 antiserum similarly to PR8-IM19/HA, whereas two mutants (PR8-IM19/ HA-135V and PR8-IM19/HA-160A) showed enhanced reactivity to this antiserum. Only one mutant (PR8-IM19/HA-160A) showed increased reactivity to MAb-1A10, and one mutant (IM19/HA-135V) showed increased reactivity to MAb-C4H4. Of note, the reactivity of PR8-IM19/HA-160A to MAb-C4H4 was 2-fold lower than that of PR8-IM19/HA to MAb-C4H4 (Fig 6E). We further generated a mutant (PR8-IM19/HA-135V160A) that contained both amino acid changes at positions 135 and 160 in the HA and found that its reactivity to H7-Re2 antiserum and MAb-1A10 was further increased (Fig 6E). The reactivity of PR8-IM19/HA-135V160A to MAb-C4H4 was 16-fold higher than that of PR8-IM19/HA to MAb-C4H4 but 4-fold lower than the cross-reactive titers of IM19/HA-135V to MAb-C4H4 (Fig 6E). These results indicate that mutations 135T and 160T in HA are important for the antigenic drift of the viruses in antigenic group II.

The V135T and A160T mutations in HA added two potential glycosylation sites in the antigenic group II viruses (S5 Fig). To investigate whether these two sites are indeed glycosylated, we performed Western blotting analysis to detect the mobility of the HA protein of different mutants. The HA of H7-Re2 and PR8-IM19/HA-135V160A moved the fastest in the gel, followed by that of PR8-IM19/HA-135V and PR8-IM19/HA-160A, and then that of PR8-IM19/HA (Fig 6F). These results indicate that the glycosylation sites at positions 133–135 and 158–160 in HA are indeed glycosylated and contributed to the antigenic drift from the H7-Re2 vaccine of the viruses in antigenic group II.

## Discussion

In the present study, we evaluated the genetic and biologic properties of H7N9 HPAIVs that were detected in poultry in China between February 2018 and December 2019. We found that the H7N9 viruses gradually lost their ability to bind to human-type receptors and were antigenically different from the H7N9 vaccine that was used in China to control H7N9 influenza in poultry. We further uncovered the genetic changes that facilitated the escape of the H7N9 viruses from the vaccine-induced immunity. On the basis of these findings, we proposed updating the H7N9 vaccine seed virus used for H7N9 influenza control in China.

The H7N9 HPAIVs emerged in Guangdong province in the beginning of 2017 and soon formed nine genotypes [7]. The present study indicates the genotype 2 viruses were still circulating in chickens in several provinces during the two years that followed the emergence of the H7N9 HPAIVs. Importantly, a virus of this genotype infected and killed a peacock in a zoo, demonstrating that the H7N9 virus can be lethal in birds other than chickens, although it still causes only mild disease in ducks.

We previously isolated H7N9 and H7N2 viruses from ducks in Fujian province; our genetic analysis indicated that those viruses were reassortants of H7N9 viruses and different unknown duck viruses, and these reassortants were reported as G8 and G9, respectively [7]. Nakayama et al. reported that an H7N3 virus that bears six genes from the G8 virus and PB2 and NA from other AIVs was detected in duck meat that was illegally carried by travelers [21]. Detection of the novel reassortant DK/FJ/SE0377/18 in this study further confirmed that the H7N9 viruses underwent complicated reassortment in ducks, and careful surveillance and control of H7 viruses in ducks will have important implications for the eradication of H7 avian influenza in China.

H7N9 HPAIVs isolated from avian species have low-to-moderate pathogenicity in mice [7], but after replication in mammalian hosts, the H7N9 influenza viruses could easily acquire more mutations––primarily the PB2 627K or PB2 701N mutation––and then become more virulent in mice [6, 22–24]. Several other amino acids in PB2, PB1, PA, and NP were reported to affect H7N9 virus replication and virulence in mammalian cells or animals [5, 25–30]. The CK/SD010/IM/19 virus is more virulent in mice than other strains in the same genotype, but all of these viruses have conserved amino acids at positions that have been reported to affect the virulence of H7N9 viruses in mammals (S1 Table), implying that some other unidentified molecular markers may have contributed to the increased virulence of CK/SD010/IM/19.

Binding to human-type receptors is a prerequisite for influenza virus to efficiently transmit from human to human and cause an influenza pandemic. Zhang et al reported that after a single round of replication in ferrets, different H3N2 AIVs could obtain mutations at position 226 or 228 in HA, and then bind human-type receptors with high affinity and become highly transmissible in ferrets [31]. Lakdawala et al. reported that replication in the soft palate of ferrets facilitates the ability of H1N1 virus to bind to long-chain α2,6-linked sialic acid [32]. AH/1/13 and CK/GD/SD008/17 bound to human-type receptors with high affinity and avian-type receptors with low affinity, which is consistent with previous studies [2, 6]. It is interesting that H7N9 HPAIVs isolated in 2018 and 2019 have increased affinity for avian-type receptors, whereas H7N9 HPAIVs isolated in 2019 have decreased affinity for human-type receptors. The L226Q mutation in HA contributes significantly to the increased affinity of H7N9 viruses for avian-type receptors; however, it remains unclear which mutation(s) contribute to the decreased affinity of H7N9 viruses for human-type receptors and which organ(s) in chickens drive the selection of H7N9 virus binding to α2,3-linked sialic acid.

Glycosylation sites that affect antigenicity have been reported in various subtypes of influenza virus [33–35]. In the present study, we found that the H7N9 viruses isolated in 2019 have different antigenicity from that of the H7N9 HPAIVs isolated in 2017 and 2018, and that two amino acid mutations––V135T and A160T––in HA, which add two glycosylation sites, appear to contribute to this antigenic drift. The fact that the mutants PR8-IM19/HA-135V and PR8-IM19/HA-160A react differently to the monoclonal antibodies MAb-C4H4 and MAb-1A10 indicates that these two glycosylation mutations may have eliminated two different antigenic epitopes. In addition to the two glycosylation sites, we also detected six other amino acid changes that are conserved in the 2019 viruses, four of which are located on the head of HA. Although none of these individual amino acid mutations changes the antigenicity, one or more of the mutations coupled with the two glycosylation sites mentioned above could further increase the degree of antigenic drift.

## Materials and methods

### Ethics statements and facility

The samples collected through active surveillance were processed in the enhanced biosafety level 2 (BSL2+) facility in the Harbin Veterinary Research Institute of the Chinese Academy of Agricultural Sciences (HVRI, CAAS), and the suspected H7N9 samples and experiments with live H7N9 viruses were conducted in the animal biosafety level 3 (ABSL3) facility in the HVRI, CAAS, which was approved for such use by the Ministry of Agriculture and Rural Affairs of China. The protocol for the animal studies was approved by the Committee on the Ethics of Animal Experiments of the HVRI, CAAS.

### Sample collection and virus isolation and identification

From February 2018 to December 2019, we collected cloacal and tracheal swab samples from 67430 birds in live poultry markets, poultry farms, and slaughterhouses in China; the two swabs from the same bird were put in one tube and counted as one sample. We also collected 3588 fresh fecal samples from wild birds in wild bird habitats and received 60 samples for disease diagnosis. All individual samples were inoculated into 10-day-old embryonated chicken eggs for 48 h at 37°C. The HA subtype was identified by using the hemagglutinin inhibition (HI) test and the NA subtype was confirmed by sequence analysis. The viruses were biologically cloned three times by limiting dilution in embryonated specific-pathogen-free (SPF) eggs, and virus stocks were maintained at −70°C.

### Genetic and phylogenetic analyses

The genomes of the H7N9 isolates were sequenced on an Applied Biosystems DNA analyzer (3500xL Genetic Analyzer, the United States). The nucleotide sequences were edited by using the Seqman module of the DNAStar package. Phylogenetic analysis was performed by using the MEGA 6.0.6 software package, implementing the neighbor-joining method, and was evaluated by 1000 bootstrap analyses; 95% sequence identity cutoffs were used to categorize the groups of each gene segment in the phylogenetic trees.

### Replication and virulence of H7N9 viruses in mice, chickens, and ducks

Six-week-old female BALB/c mice (Beijing Experimental Animal Center, Beijing, China) were mildly anesthetized with CO_2_ and then inoculated intranasally (i.n.) with 10^6^ 50% egg infective doses (EID_50_) of H7N9 virus in a volume of 50 μl. Three mice in each group were euthanized on Day 3 post-inoculation (p.i.) and their organs, including brains, nasal turbinates, spleen, kidneys, and lungs, were collected for virus titration. The remaining five mice were monitored daily for weight loss and mortality for up to two weeks. The 50% mouse-lethal dose (MLD_50_) was determined by inoculating groups of five mice i.n. with 10-fold serial dilutions containing 10^3^–10^6^ EID_50_ of test virus and were monitored for 14 days for weight loss and mortality.

To determine the replication and virulence of the H7N9 viruses in chickens or ducks, groups of 11 six-week-old SPF White Leghorn chickens or groups of eight 3-week-old female SPF ducks (Shaoxing shelduck, a local breed) (Harbin Experimental Animal Center, Harbin, China) seronegative for avian influenza viruses were inoculated i.n. with 0.1 ml of 10^6^ EID_50_ of test viruses. Pharyngeal and cloacal swabs were collected from all birds on day 3 p.i. and then three birds in each group were killed and their organs (lungs, heart, liver, spleen, kidneys, pancreas, and brain) were collected for virus titration in eggs. The remaining eight chickens and five ducks were observed for two weeks.

### Receptor-binding analysis

Receptor specificity was analyzed by use of a solid-phase direct binding assay as described previously using two different glycopolymers: α-2, 3-sialylglycopolymer [Neu5Acα2-3Galb1-4GlcNAcb1-pAP (para-aminophenyl)-alpha-polyglutamic acid (α-PGA)] and the α-2, 6-sialylglycopolymer [Neu5Acα2-6Galb1-4GlcNAcb1-pAP (para-aminophenyl)-alpha-polyglutamic acid (α-PGA)] [36, 37]. Chicken antiserum induced by a DNA vaccine that expressing the HA gene of the CK/GX/SD098/17 (H7N9) virus and a horseradish peroxidase (HRP)-conjugated goat-anti-chicken antibody (Sigma-Aldrich, St. Louis, MO, USA) were used in this assay.

### Antisera and antigenic analyses

Antigenic analysis of H7N9 viruses was performed by using HI tests with antisera generated in chickens against different inactivated viruses, including H7-Re2, CK/LN/SD014/2018, and CK/IM/SD010/2019. The HI assay was performed as previously described [38]. Antigenic maps of the viruses were obtained by using Antigenic Cartography software (http://www.antigenic-cartography.org/).

### Vaccine

The commercial trivalent H5/H7 inactivated oil-emulsified vaccine based on the H5 Re-11, Re-12, and H7-Re2 viruses was developed as previously reported by Zeng et al. [12] and was provided by Harbin Weike Biotechnology Development Company (Harbin, China). The Re-11 strain contains the HA gene from DK/GZ/S4184/17 (H5N6) (clade 2.3.4.4d) and the NA gene from the CK/GZ/4/13 virus (clade 2.3.4.4b), the Re-12 strain contains the HA and NA genes from CK/LN/SD007/17 (H5N1) (clade 2.3.2.1d), and the H7-Re2 strain contains the HA and NA genes from the HPAI H7N9 virus CK/GX/SD098/17. The internal genes of these vaccine seed viruses are from PR8 virus, and the cleavage site of the HA gene of these vaccine seed viruses was modified to reduce its virulence as reported previously [7, 39].

### Challenge study in chickens

Groups of 10 three-week-old female SPF chickens were inoculated intramuscularly with 0.3 ml of the vaccine or with an equal volume of PBS as a control. Three weeks post-vaccination, antisera were collected from the birds and the HI titers were measured by using the homologous vaccine strain and challenge viruses. The chickens were then challenged i.n. with 10^5^EID_50_ of H7N9 viruses. Pharyngeal and cloacal swabs were collected from all surviving birds on Days 3 and 5 post-challenge (p.c.) and titrated in eggs. The birds were observed for signs of disease and death for two weeks.

### 3D structural analysis of the HA protein

The 3D structure of the HA protein was obtained by using SWISS-MODEL; the image of the HA protein was drawn by using Pymol software as described previously [40].

### Virus rescue

The PR8-IM19 reassortant virus, containing the HA and NA genes from an H7N9 virus (CK/IM/SD010/2019 (IM19)) and the six internal genes from the PR8 virus, was rescued by reverse genetics as previously reported [39]. Using the PR8-IM19 reassortant virus as a model, mutations were introduced into the HA gene by PCR using the QuickChange Site-Directed Mutagenesis kit. Primer sequences are available upon request. The rescued viruses were fully sequenced to ensure the absence of unwanted mutations.

### Western blot analysis

Viruses were concentrated by ultracentrifugation and purified by sucrose gradient centrifugation as described previously [34,41]. The virus samples were analyzed by SDS-PAGE and Western blotting. The chicken antisera induced by the pCAGGS-HA (HA gene obtained from the CK/GX/SD098/2017 virus) were used as the primary antibody, and IRDyeTM700DX-conjugated rabbit anti-chicken antiserum was used as the secondary antibody (Sigma).

## Supporting information

S1 Fig Cases of human infections with H7N9 viruses. The total number of H7N9 human cases as of 31 January 2021. The dashed lines indicate October 1st of each year. The red arrow indicates when H5/H7 vaccine administration to poultry was initiated in China.
(TIF)

S2 Fig Phylogenetic analysis of the PB2 and PB1 genes of H7N9 viruses. The phylogenetic trees of PB2 **(A)** and PB1 **(B)** were rooted to A/chicken/Rostock/45/1934 (H7N1). The viruses sequenced in this study are shown in red and blue in the phylogenetic trees.
(TIF)

S3 Fig Phylogenetic analysis of the PA and NP genes of H7N9 viruses. The phylogenetic trees of PA **(A)** and NP **(B)** were rooted to A/chicken/Rostock/45/1934 (H7N1). The viruses sequenced in this study are shown in red and blue in the phylogenetic trees.
(TIF)

S4 Fig Phylogenetic analysis of the M and NS genes of H7N9 viruses. The phylogenetic trees of M **(A)** and NS **(B)** were rooted to A/chicken/Rostock/45/1934 (H7N1). The viruses sequenced in this study are shown in red and blue in the phylogenetic trees.
(TIF)

S5 Fig Potential N-glycosylation sites in the HA of H7N9 viruses. The dots show the potential N-glycosylation sites at the corresponding positions in the HA protein of H7N9 viruses. Abbreviations: CK, chicken; DK, duck; PCK, peacock; AH, Anhui; FJ, Fujian; GX, Guangxi; HeB, Hebei; IM, Inner Mongolia; LN, Liaoning; NX, Ningxia; SaX, Shaanxi; SX, Shanxi.
(TIF)

S1 Table Amino acids in H7N9 viruses that contribute to increased binding to human-type receptors, replication, virulence, or transmission in mammals. (DOCX)

S2 Table Antigenic analysis of H7N9 viruses. (DOCX)

S3 Table Amino acid differences in the antigenic site in the HA1 protein of H7N9 viruses. (DOCX)
