# Supplementary figures and images for: Genetic and biological properties of H7N9 avian influenza viruses detected after application of the H7N9 poultry vaccine in China
Source: PLoS Pathog. 2021 Apr 27;17(4):e1009561. doi: 10.1371/journal.ppat.1009561 (PMC8104392; doi:10.1371/journal.ppat.1009561)

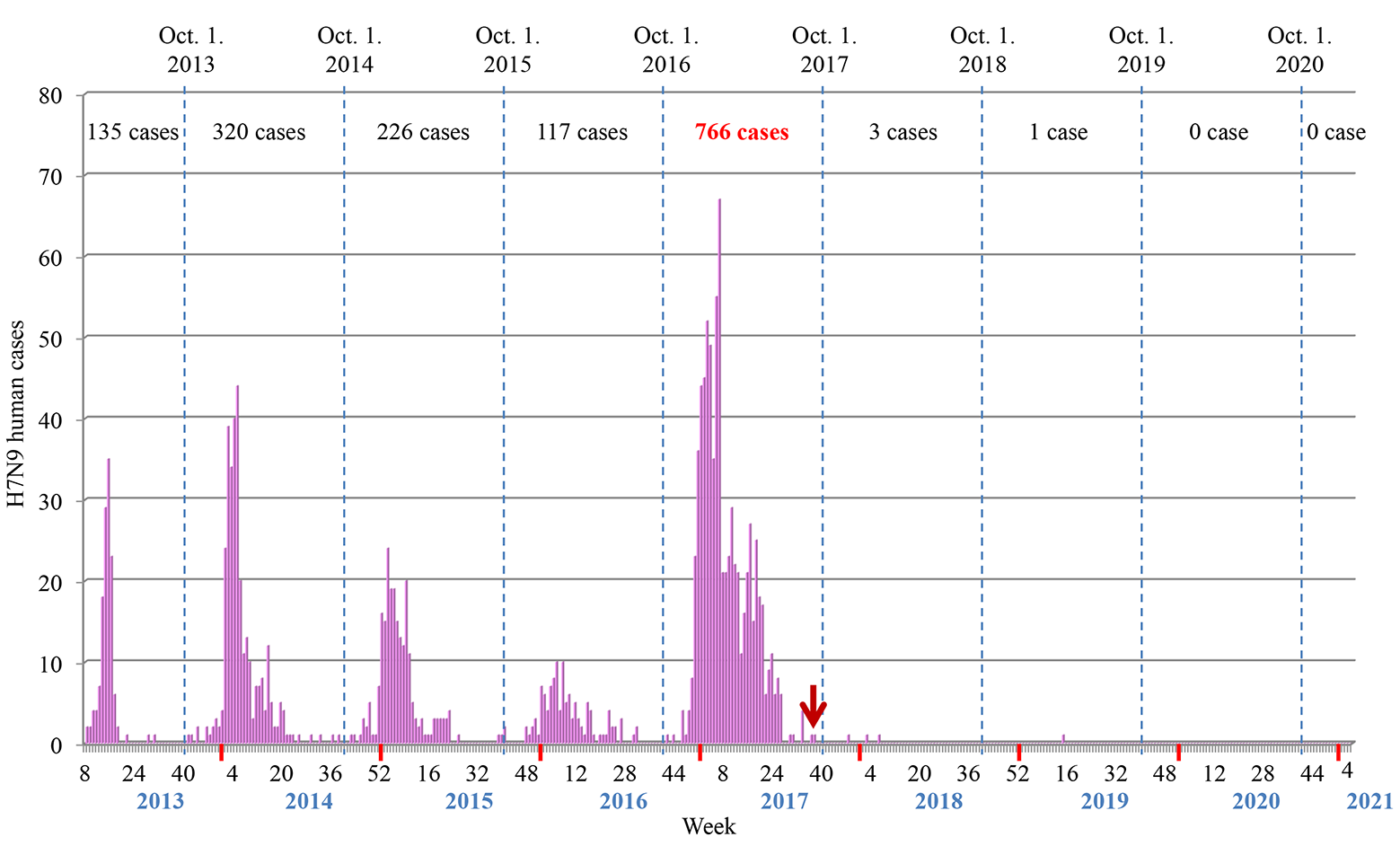

Supplement: S1 Fig — The total number of H7N9 human cases as of 31 January 2021. The dashed lines indicate October 1st of each year. The red arrow indicates when H5/H7 vaccine administration to poultry was initiated in China. (TIF) [file ppat.1009561.s001.tif]

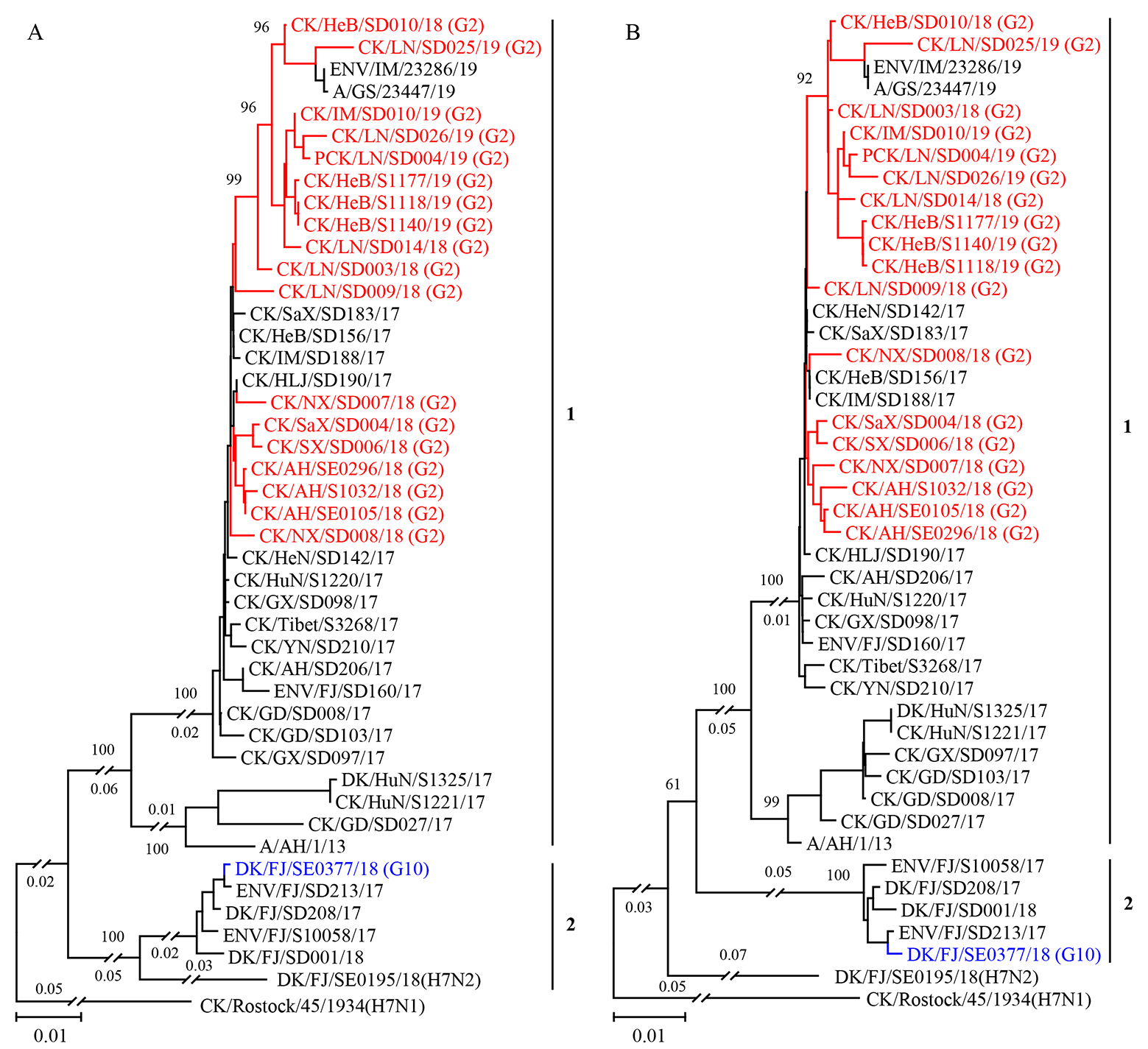

Supplement: S2 Fig — The phylogenetic trees of PB2 (A) and PB1 (B) were rooted to A/chicken/Rostock/45/1934 (H7N1). The viruses sequenced in this study are shown in red and blue in the phylogenetic trees. (TIF) [file ppat.1009561.s002.tif]

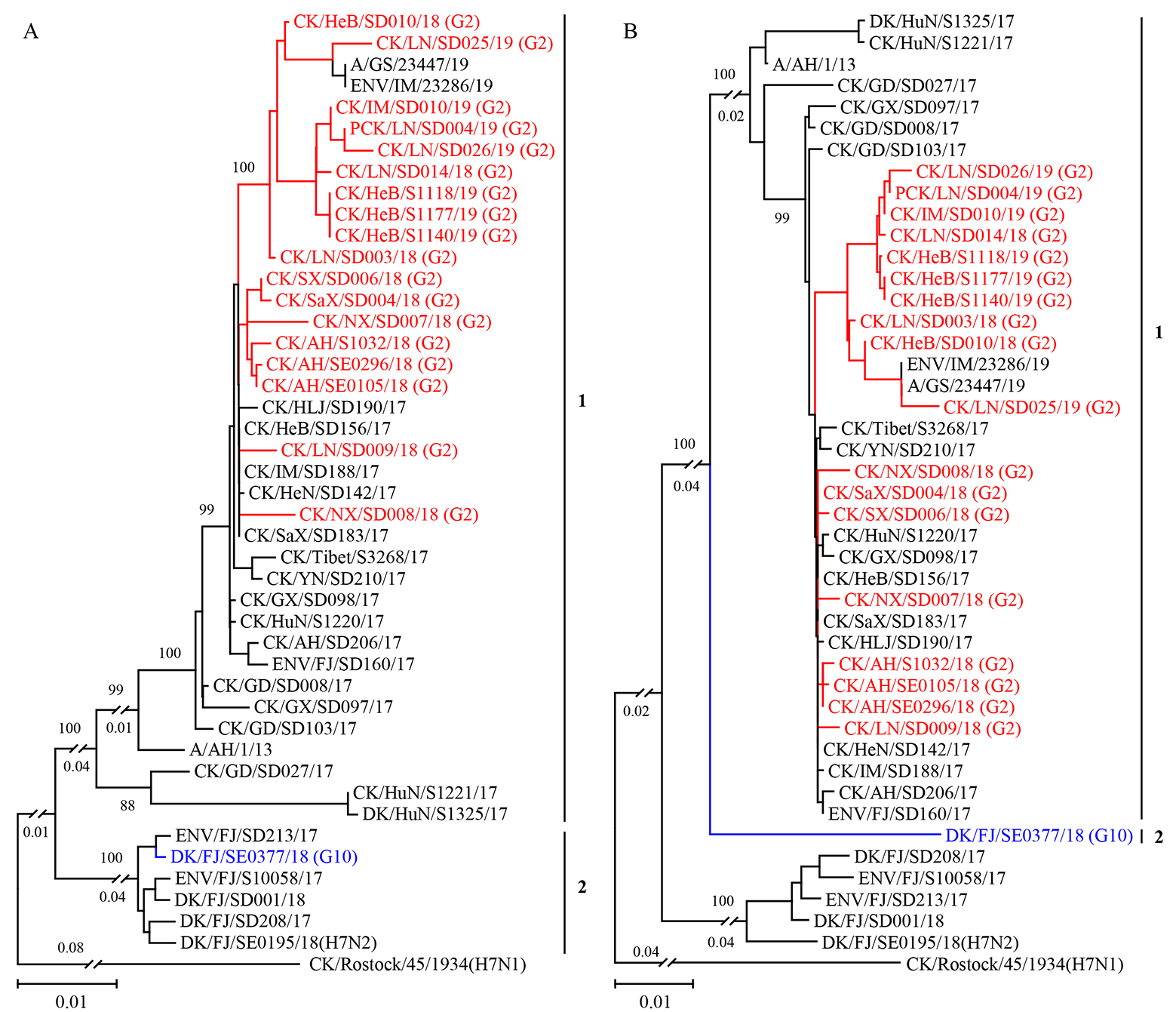

Supplement: S3 Fig — The phylogenetic trees of PA (A) and NP (B) were rooted to A/chicken/Rostock/45/1934 (H7N1). The viruses sequenced in this study are shown in red and blue in the phylogenetic trees. (TIF) [file ppat.1009561.s003.tif]

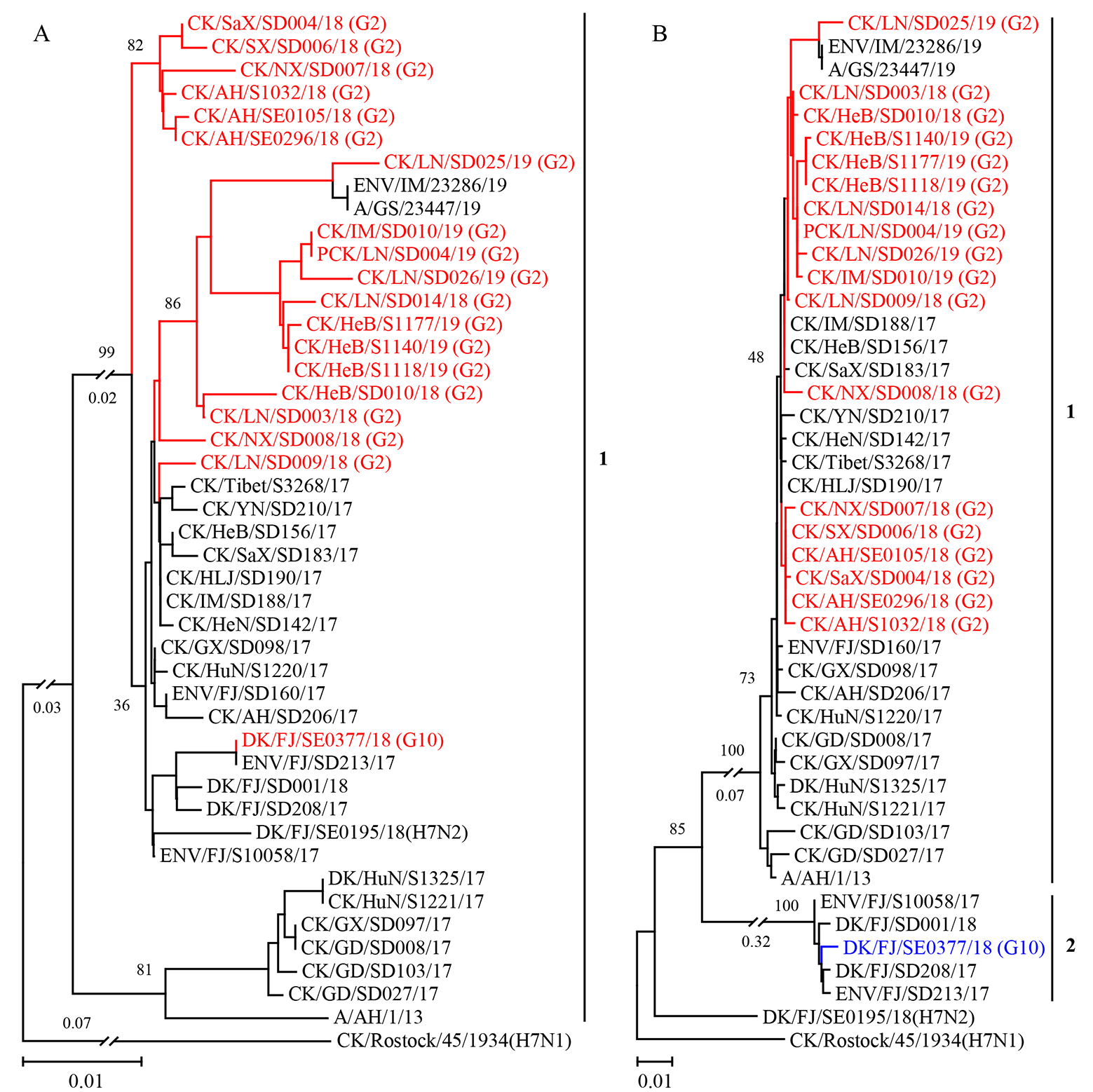

Supplement: S4 Fig — The phylogenetic trees of M (A) and NS (B) were rooted to A/chicken/Rostock/45/1934 (H7N1). The viruses sequenced in this study are shown in red and blue in the phylogenetic trees. (TIF) [file ppat.1009561.s004.tif]

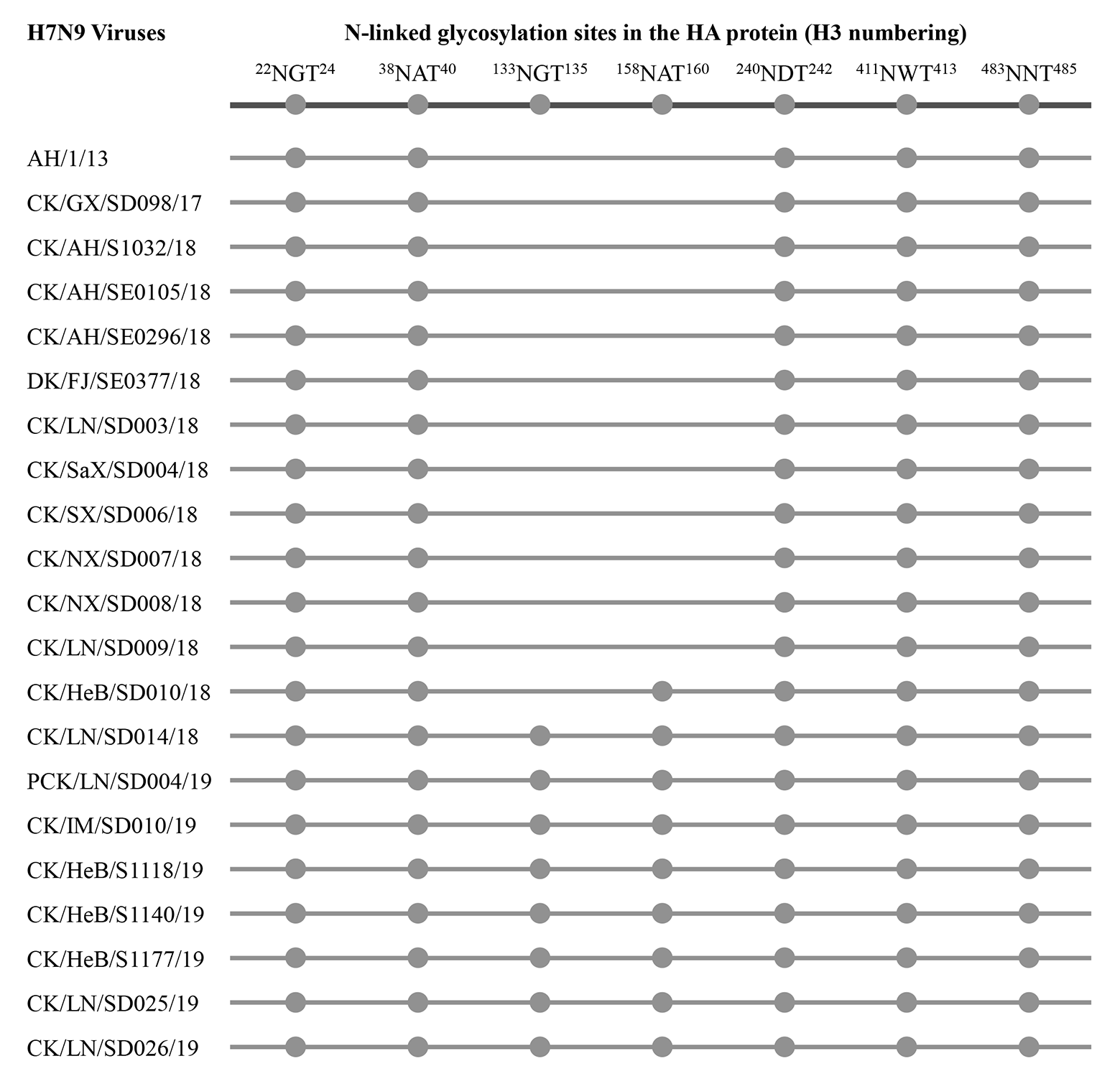

Supplement: S5 Fig — The dots show the potential N-glycosylation sites at the corresponding positions in the HA protein of H7N9 viruses. Abbreviations: CK, chicken; DK, duck; PCK, peacock; AH, Anhui; FJ, Fujian; GX, Guangxi; HeB, Hebei; IM, Inner Mongolia; LN, Liaoning; NX, Ningxia; SaX, Shaanxi; SX, Shanxi. (TIF) [file ppat.1009561.s005.tif]
