# Supplementary material for: Genetic and biological properties of H7N9 avian influenza viruses detected after application of the H7N9 poultry vaccine in China
Source: PLoS Pathog. 2021 Apr 27;17(4):e1009561. doi: 10.1371/journal.ppat.1009561 (PMC8104392; doi:10.1371/journal.ppat.1009561)
Supplement: S1 Table — (DOCX) [file ppat.1009561.s006.docx]

**S1 Table. Amino acids in H7N9 viruses that contribute to increased binding to human-type receptors, replication, virulence, or transmission in mammals.**

| Virus | Mutations in HA that increase affinity to human-type receptors [5, 6, 32] | | | |  | Mutations that increase the replication, virulence, or transmission of avian influenza viruses in mammalian hosts [5-6, 23-31] | | | | | | | | | | |
| --- | --- | --- | --- | --- | --- | --- | --- | --- | --- | --- | --- | --- | --- | --- | --- | --- |
|  |  |  |  |  |  | PB2 | | |  | PB1 |  | PA | |  | NP | |
|  | T160A | G186V | Q226L | G228S |  | I292V | K526R | E627K |  | I368V |  | V100A | K356R |  | Combination of 286A and 437T |  |
| AH/1/13 | A | V | L | G |  | V | K | K |  | V |  | A | R |  | 286A, 437T |  |
| CK/GD/SD008/17 | A | V | L | G |  | I | R | E |  | V |  | A | R |  | 286A, 437T |  |
| CK/GX/SD098/17 | A | V | Q | G |  | I | R | E |  | V |  | A | R |  | 286V, 437M |  |
| CK/AH/S1032/18 | A | V | Q | G |  | I | R | E |  | V |  | A | R |  | 286A, 437T |  |
| CK/AH/SE0105/18 | A | V | Q | G |  | I | R | E |  | V |  | A | R |  | 286A, 437T |  |
| CK/AH/SE0296/18 | A | V | Q | G |  | I | R | E |  | V |  | A | R |  | 286A, 437T |  |
| DK/FJ/SE0377/18 | A | V | Q | G |  | I | K | E |  | I |  | F | K |  | 286A, 437T |  |
| CK/LN/SD003/18 | A | V | Q | G |  | I | R | E |  | V |  | A | R |  | 286A, 437T |  |
| CK/SaX/SD004/18 | A | V | Q | G |  | I | R | E |  | V |  | A | R |  | 286A, 437T |  |
| CK/SX/SD006/18 | A | V | Q | G |  | I | R | E |  | V |  | A | R |  | 286A, 437T |  |
| CK/NX/SD007/18 | A | V | Q | G |  | I | R | E |  | V |  | A | R |  | 286A, 437T |  |
| CK/NX/SD008/18 | A | V | Q | G |  | I | R | E |  | V |  | A | R |  | 286A, 437T |  |
| CK/LN/SD009/18 | A | V | Q | G |  | I | R | E |  | V |  | A | R |  | 286A, 437T |  |
| CK/HeB/SD010/18 | T | V | Q | G |  | I | R | E |  | V |  | T | R |  | 286A, 437T |  |
| CK/LN/SD014/18 | T | V | Q | G |  | I | R | E |  | V |  | A | R |  | 286A, 437T |  |
| PCK/LN/SD004/19 | T | V | Q | G |  | I | R | E |  | V |  | A | R |  | 286A, 437T |  |
| CK/IM/SD010/19 | T | V | Q | G |  | I | R | E |  | V |  | A | R |  | 286A, 437T |  |
| CK/HeB/S1118/19 | T | V | Q | G |  | I | R | E |  | V |  | A | R |  | 286A, 437T |  |
| CK/HeB/S1140/19 | T | V | Q | G |  | I | R | E |  | V |  | A | R |  | 286A, 437T |  |
| CK/HeB/S1177/19 | T | V | Q | G |  | I | R | E |  | V |  | A | R |  | 286A, 437T |  |
| CK/LN/SD025/19 | T | V | Q | G |  | I | R | E |  | V |  | T | R |  | 286A, 437T |  |
| CK/LN/SD026/19 | T | V | Q | G |  | I | R | E |  | V |  | A | R |  | 286A, 437T |  |
