# Supplementary material for: Genetic and biological properties of H7N9 avian influenza viruses detected after application of the H7N9 poultry vaccine in China
Source: PLoS Pathog. 2021 Apr 27;17(4):e1009561. doi: 10.1371/journal.ppat.1009561 (PMC8104392; doi:10.1371/journal.ppat.1009561)
Supplement: S2 Table — (DOCX) [file ppat.1009561.s007.docx]

**S2 Table. Antigenic analysis of H7N9 viruses.**

| Virus | Cross-reactive HI antibody titers of chicken antisera against different viruses^a^ | | |
| --- | --- | --- | --- |
|  | H7-Re2 | CK/LN/SD014/18 | CK/IM/SD010/19^b^ |
| H7-Re2 | 512 | 128 | 128 |
| CK/GX/SD098/17 | 256 | 128 | 128 |
| CK/LN/SD014/18 | 32 | 512 | 256 |
| CK/IM/SD010/19 | 16 | 256 | 512 |
| CK/AH/S1032/18 | 256 | 64 | 128 |
| CK/AH/SE0105/18 | 128 | 64 | 64 |
| CK/AH/SE0296/18 | 256 | 64 | 64 |
| DK/FJ/SE0377/18 | 128 | 64 | 32 |
| CK/LN/SD003/18 | 256 | 64 | 64 |
| CK/SaX/SD004/18 | 128 | 64 | 64 |
| CK/SX/SD006/18 | 256 | 64 | 64 |
| CK/NX/SD007/18 | 128 | 64 | 64 |
| CK/NX/SD008/18 | 128 | 64 | 32 |
| CK/LN/SD009/18 | 256 | 64 | 64 |
| CK/HeB/SD010/18 | 256 | 64 | 128 |
| PCK/LN/SD004/19 | 32 | 256 | 256 |
| CK/HeB/S1118/19 | 16 | 256 | 256 |
| CK/HeB/S1140/19 | 16 | 256 | 256 |
| CK/HeB/S1177/19 | 16 | 256 | 256 |
| CK/LN/SD025/19 | 16 | 128 | 128 |
| CK/LN/SD026/19 | 16 | 128 | 256 |

^a^ Homologous titer is underlined.

^b^ Antisera against the H7-Re2 vaccine strain and representative H7N9 viruses were generated in chickens.
