# Supplementary material for: Genetic and biological properties of H7N9 avian influenza viruses detected after application of the H7N9 poultry vaccine in China
Source: PLoS Pathog. 2021 Apr 27;17(4):e1009561. doi: 10.1371/journal.ppat.1009561 (PMC8104392; doi:10.1371/journal.ppat.1009561)
Supplement: S3 Table — (DOCX) [file ppat.1009561.s008.docx]

**S3 Table. Amino acid differences in the antigenic site in the HA1 protein of H7N9 viruses.**

| Virus | Amino acid positions in the HA1 protein (H3 numbering) | | | | | | | |
| --- | --- | --- | --- | --- | --- | --- | --- | --- |
|  | 19 | 81 | 88 | 126 | 135 | 145 | 160 | 310 |
| CK/GX/SD098/17 | A | E | I | T | V | S | A | K |
| CK/AH/S1032/18 | A | E | I | T | V | S | A | K |
| CK/AH/SE0105/18 | A | E | I | T | V | S | A | K |
| CK/AH/SE0296/18 | A | E | I | T | V | S | A | K |
| DK/FJ/SE0377/18 | A | E | I | T | V | S | A | K |
| CK/LN/SD003/18 | S | E | V | T | V | S | A | R |
| CK/SaX/SD004/18 | A | E | I | T | V | S | A | K |
| CK/SX/SD006/18 | A | E | I | T | V | S | A | K |
| CK/NX/SD007/18 | A | E | I | T | V | S | A | K |
| CK/NX/SD008/18 | A | E | I | T | V | S | A | K |
| CK/LN/SD009/18 | A | E | I | T | A | S | A | K |
| CK/HeB/SD010/18 | S | E | V | T | V | S | T | R |
| CK/LN/SD014/18 | S | K | V | K | T | S | T | R |
| PCK/LN/SD004/19 | S | K | V | K | T | P | T | R |
| CK/IM/SD010/19 | S | K | V | K | T | P | T | R |
| CK/HeB/S1118/19 | S | K | V | K | T | P | T | R |
| CK/HeB/S1140/19 | S | K | V | K | T | P | T | R |
| CK/HeB/S1177/19 | S | K | V | K | T | P | T | R |
| CK/LN/SD025/19 | S | K | V | K | T | P | T | R |
| CK/LN/SD026/19 | S | K | V | K | T | P | T | R |
